# Addressing prioritization in healthcare amidst a global pandemic

**DOI:** 10.1177/08404704211002539

**Published:** 2021-04-05

**Authors:** Craig Mitton, Cam Donaldson, Francois Dionne, Stuart Peacock

**Affiliations:** 1 School of Population and Public Health, University of British Columbia, Vancouver, British Columbia, Canada.; 2 Centre for Clinical Epidemiology and Evaluation, Vancouver Coastal Health Research Institute, Vancouver, British Columbia, Canada.; 3 Glasgow Caledonian University, Glasgow, Scotland.; 4 Faculty of Health Sciences, Simon Fraser University, Vancouver, British Columbia, Canada.; 5 Canadian Centre for Applied Research in Cancer Control, BC Cancer, Vancouver, British Columbia, Canada.; 6 Cancer Control Research, BC Cancer, Vancouver, British Columbia, Canada.

## Abstract

Trade-offs abound in healthcare yet depending on where one stands relative to the stages of a pandemic, choice making may be more or less constrained. During the early stages of COVID-19 when there was much uncertainty, healthcare systems faced greater constraints and focused on the singular criterion of “flattening the curve.” As COVID-19 progressed and the first wave diminished (relatively speaking depending on the jurisdiction), more opportunities presented for making explicit choices between COVID and non-COVID patients. Then, as the second wave surged, again decision makers were more constrained even as more information and greater understanding developed. Moving out of the pandemic to recovery, choice making becomes paramount as there are no set rules to lean back into historical patterns of resource allocation. In fact, the opportunity at hand, when using explicit tools for priority setting based on economic and ethical principles, is significant.

## Introduction

Setting priorities and allocating scarce resources is an activity that healthcare decision makers have always had to address, both in Canada and elsewhere.^1^ Limited or constrained budgets lead to the inevitability of choices having to be made, usually at the margin, about what to fund and what not to fund.^2^ The process of making these choices is referred to as priority setting. On the back of examining choices and assessing relative value of both clinical and non-clinical options, decision makers are charged with determining where resources are best placed.

During a pandemic, such choices and allocation decisions are under even greater scrutiny as the demand for urgent services is significantly increased. Throughout COVID-19, it has been very clear to the public that healthcare organizations are making trade-offs with respect to which patients will receive services and how quickly certain patients will be treated.^3^ There should be nothing surprising about this in and of itself. That is, as we have just stated, choice making is a global phenomenon in healthcare. One might suggest that this is the *raison d’etre* of healthcare managers. The pandemic, and recovery from it, simply highlight this fact.

Having said that, we would be quick to acknowledge that, at certain points during the COVID-19 pandemic, there has been less flexibility in making choices because of limited knowledge about the virus, at first, and then, in the second wave, because of the rapid spread of the virus. But, looking beyond the current pandemic to recovery, more attention may well be given to the process of decision making specifically around relative value assessment and the trade-offs that ensue in part because of the need for catching up on delayed interventions.

The aim of this short article is to highlight some of the challenging issues around trade-offs and then to review relevant tools for priority setting and resource allocation that can be part of managing the recovery in the wake of the pandemic.

## Trade-offs everywhere

During the first wave of COVID in the Spring of 2020, there was much uncertainty. It was not clear what the R value (the number of people, on average, that one infected person will pass the virus on to) was. Nor was there clarity on the underlying biological mechanisms, the forms of transmission, how best to treat this disease or expected outcomes. In this state, decision makers fell back on the precautionary principle and many jurisdictions went into lockdown. In the health system, capacity was freed up through the cancellation of hundreds of thousands of elective surgical procedures across Canada, initially for reasons of safety. As the first wave subsided and more information became available, different decisions could be made on public health measures. Individual and community “choices” were emphasized including physical distancing, minimizing travel, and mask wearing. From a health system perspective, there was also, at times, room for greater choice making. For example, over the Summer and early Fall 2020, there could be less of a focus on “flattening the curve” and more on taking otherwise freed up capacity and allocating resources back toward non-COVID patients.

As the second wave hit and the health system faced increasing pressure, once again capacity issues were identified and there was less ability for decision makers to make choices. Having said that, during the second wave, there was more information at hand and as such, more nuanced and evidence-based decisions could be made when applying broad policy tools such as lockdowns. Also, vaccines were developed with varying, but for the most part, high levels of effectiveness at reducing symptoms (noting longer term evidence of their impact on mitigating transmission is still required).^4^ This further enabled decision makers to address choice making directly and provided an opportunity to be explicit in determining how best limited resources would be allocated. In this, one may also reflect, critically, on the unintended consequences at the different phases of the pandemic as these can inform choice making going forward. For example, increases in domestic violence, isolation leading to mental health challenges, higher rates of overdose due to illicit drug toxicity, and challenges of social well-being for children unable to see their friends, among other things, should all be assessed.

From an economic perspective, the fundamental principle of opportunity cost is at play in almost every decision made in the health system. As there are limited resources, funding of one group leads to some benefit lost in not funding another group. Despite the contention that healthcare managers have the ability to make choices, in the early days of the pandemic, this was curtailed as economy and health went hand in hand and non-COVID care was cut back.^5^ Systems with greater capacity already built in seemed to do better, whereas elsewhere the needed capacity had to be gotten from “somewhere.”^5^ As our understanding of the disease increased and effective vaccines were developed, health systems have begun to prepare for recovery and future resilience. Moving forward, questions of trade-offs should be addressed explicitly. The good news is there are readily available priority setting tools that can be used to assist decision makers in these often-complex and politically charged waters.

## Priority setting tools

**Figure 1.**
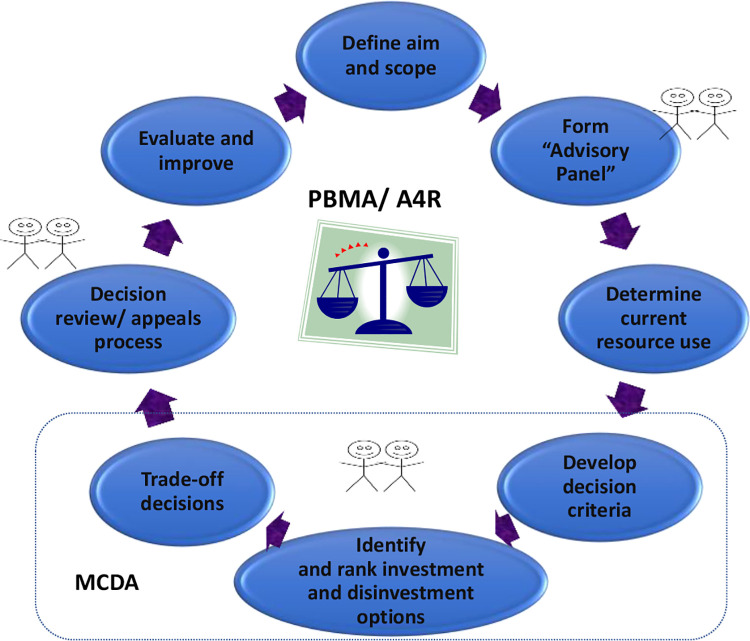
Process for priority setting and resource allocation in healthcare. A4R indicates Accountability for Reasonableness; MCDA, multi-criteria decision analysis; key areas for public involvement indicated by stick figures; PBMA, program budgeting and marginal analysis.

Use of a formal approach to priority setting and resource allocation would typically involve the eight steps illustrated in Figure 1.^6^ There is room within this type of framework for the application of high-quality evidence, including the form often produced in health technology assessments, and further, there are multiple opportunities for substantive public engagement.^7^ Program Budgeting and Marginal Analysis (PBMA) and Accountability for Reasonableness are two frameworks that have been used in concert for many years to assist decision makers in determining how best to allocate scarce resources.^8^

Within an explicit approach to priority setting, relative value of options can be assessed using Multi-Criteria Decision Analysis enabling decision makers to determine explicitly the benefit gain of competing alternatives for the limited resources.^9^ Clear and transparent criteria, weighted to reflect their relative importance, are an effective means to reflect preferences from different stakeholder groups with regards to healthcare management. Determination of those criteria can inform decisions on who gets the limited resources as well as the magnitude of costs and benefits, thus providing a clear understanding of the trade-offs involved along with equity implications.^10,11^

With routine use of a prioritization framework as presented herein (see again Figure 1), decision makers are able to determine not only what the quantifiable trade-offs are but also how, on an ongoing basis, resources should best be allocated.^7,12^ There are no “set” results that come out of the application of these tools and indeed it is almost certain that different jurisdictions will come to different decisions, reflecting population make-up, resources availability, and societal preferences. There are many examples of this type of approach in the health policy and management literature, with our own work spanning over 100 organizations.

One recent case study was in a large urban hospital in Ontario, which sought to mitigate some financial pressure as well as optimize existing spend through resource reallocation. This was done pre-pandemic, but the challenges faced then were the same as will be faced in pandemic recovery except that in pandemic recovery the financial pressures will only be greater because of the new required spending. The PBMA process was applied as per the steps in Figure 1. A multidisciplinary priority setting steering committee was struck, decision criteria (and their weights) were selected, proposals for both investment and disinvestment were developed and rated, and decisions were made based on explicit evaluation of the proposals against the criteria. Twelve criteria were identified for this process including access, health gain, equity, client experience, and innovation, to name a few. In addition, a formal rating tool was utilized by the steering committee in coming up with resource allocation recommendations. In all, the process took about eight months with decisions incorporated into the annual budget process.

In the wake of a pandemic, as health systems get “back on their feet,” it becomes critical to be explicit in the analysis of opportunity costs because there will be significant reallocation of resources. Examples of reallocations across the health system include the need to invest in ongoing public health vaccination and track and trace programs; strengthening PPE supply chains; critical vaccine production capacity; and health human resources needed to manage endemic COVID-19. These investments will need to be balanced against potential disinvestments elsewhere in the system, for example, longer elective surgery wait times. Each organization across the country will need to assess trade-offs and determine to what extent specific reallocations make sense. One particular area that was substantially rocked through the pandemic is long-term care. These organizations will need to assess trade-offs within their limited budgets. The type of approach described in this article can be applied to walk a management team through the steps for reallocation, balancing important criteria such as patient safety, caregiver safety, patient experience and well-being, and access to care, to name just a few. Having such a framework in place can lead to greater accountability in this sector.

An important corollary is the degree of public involvement in healthcare priority setting. There is good evidence to show that a broader set of constituents can be engaged in healthcare decision making.^13,14^ In fact, it is plausible that, in the recovery phase of a pandemic, public consultation is even more paramount. In addition, building on this notion of public engagement would be to determine values around preparedness for future pandemics. How much should the possibility of the next pandemic be considered relative to short or even longer term non-pandemic goals? Along these lines, thinking on portfolio approaches to investment may well help in determining how much to allocate in preparing for future pandemics.^15^ The Canadian public has shown remarkable trust in the health system; it is vital that this trust is preserved through meaningful, ongoing, public engagement.

One final point is warranted. Although the focus here is on the application of an evidence-informed process for priority setting within healthcare, of course, the implications of public policy including restrictions placed on individuals and communities go well beyond healthcare and enter “whole of government.” The most obvious of these implications is the economic impact as the Gross Domestic Product has dropped in Canada as well as elsewhere since the start of the COVID-19 pandemic. Other examples of economic trade-offs include income support for workers who have been laid off due to COVID-19 restrictions; funding for sick leave for individuals who have to self-isolate; and, child care costs for parents during school closures. As such, we would suggest that the type of thinking behind the approach to priority setting put forward here is also highly applicable in determining the cross-sectoral impacts. Recent work from the United Kingdom and Canada indicates how this type of thinking can inform decision making.^16^

## Conclusion

In summary, we are strong proponents for the use of explicit, evidence-informed tools for setting priorities and allocating resources in healthcare. This sentiment has recently been echoed by others in the healthcare management space in calling for more transparency in decision making.^17^ Although such tools are always relevant, their application can be severely limited during a pandemic due to extreme constraints on choice making. But, as we move into pandemic recovery, such tools become highly relevant because the range of possible choices is broader and there is no requirement to go back to the same resource allocation patterns. We would implore government agencies to look seriously at these tools as they can assist in determining the relative value of trade-offs and optimizing use of the limited resources at a time when there will be important choices to be made.
